# Sexual violence from police and HIV risk behaviours among HIV-positive women who inject drugs in St. Petersburg, Russia – a mixed methods study

**DOI:** 10.7448/IAS.19.4.20877

**Published:** 2016-07-18

**Authors:** Karsten Lunze, Anita Raj, Debbie M Cheng, Emily K Quinn, Fatima I Lunze, Jane M Liebschutz, Carly Bridden, Alexander Y Walley, Elena Blokhina, Evgeny Krupitsky, Jeffrey H Samet

**Affiliations:** 1 Department of Medicine, Boston University School of Medicine, Boston, MA, USA; 2 Division of Global Public Health, University of California, San Diego, CA, USA; 3 Department of Biostatistics, Boston University School of Public Health, Boston, MA, USA; 4 Boston Children's Hospital, Boston, MA, USA; 5 Boston Medical Center, Boston, MA, USA; 6 Laboratory of Clinical Psychopharmacology of Addictions, Valdman Institute of Pharmacology, St. Petersburg First Pavlov State Medical University, St. Petersburg, Russia; 7 Department of Addictions, St.-Petersburg Bekhterev Psychoneurological Research Institute, St. Petersburg, Russia

**Keywords:** sexual violence, gender-based violence, human rights, police involvement, People living with HIV/AIDS (PLHA), injection drug use, key populations, Russian Federation

## Abstract

**Introduction:**

Police violence against people who inject drugs (PWID) is common in Russia and associated with HIV risk behaviours. Sexual violence from police against women who use drugs has been reported anecdotally in Russia. This mixed-methods study aimed to evaluate sexual violence from police against women who inject drugs via quantitative assessment of its prevalence and HIV risk correlates, and through qualitative interviews with police, substance users and their providers in St. Petersburg, Russia.

**Methods:**

Cross-sectional analyses with HIV-positive women who inject drugs (*N*=228) assessed the associations between sexual violence from police (i.e. having been forced to have sex with a police officer) and the following behaviours: current drug use, needle sharing and injection frequency using multiple regression models. We also conducted in-depth interviews with 23 key informants, including PWID, police, civil society organization workers, and other stakeholders, to explore qualitatively the phenomenon of sexual violence from police in Russia and strategies to address it. We analyzed qualitative data using content analysis.

**Results:**

Approximately one in four women in our quantitative study (24.1%; 95% CI, 18.6%, 29.7%) reported sexual violence perpetrated by police. Affected women reported more transactional sex for drugs or money than those who were not; however, the majority of those reporting sexual violence from police were not involved in these forms of transactional sex. Sexual violence from police was not significantly associated with current drug use or needle sharing but with more frequent drug injections (adjusted incidence rate ratio 1.43, 95% CI 1.04, 1.95). Qualitative data suggested that sexual violence and coercion by police appear to be entrenched as a norm and are perceived insurmountable because of the seemingly absolute power of police. They systematically add to the risk environment of women who use drugs in Russia.

**Conclusions:**

Sexual violence from police was common in this cohort of Russian HIV-positive women who inject drugs. Our analyses found more frequent injection drug use among those affected, suggesting that the phenomenon represents an underappreciated human rights and public health problem. Addressing sexual violence from police against women in Russia will require addressing structural factors, raising social awareness and instituting police trainings that protect vulnerable women from violence and prevent HIV transmission.

## Introduction

People who inject drugs (PWID) are a key population at increased risk of HIV transmission through unsafe drug injection practices with HIV-contaminated needles. In the Russian Federation (Russia), injection drug use (IDU) is the main route of transmission of a dramatically expanding HIV epidemic at risk of bridging from high-risk groups such as PWID or sex workers to the general population. Close to one million people in Russia are HIV-positive [1]. Simultaneously, the number of people estimated to be injecting drugs has increased, in the past five years alone by more than half a million [2]. In Russia, 2.3% of the adult population (1.8 million people) inject drugs and 14.4% of PWID are HIV-positive, making the country one of the most affected by IDU and HIV [3].

In addition to personal behaviours (e.g. drug injection or sex practices), structural factors in the environments in which PWID live determine HIV transmission risks. These factors “produce” a risk environment [4]. For example, in previous Russian work involving 582 PWID, police violence against HIV-positive PWID was common and had adverse effects on HIV transmission risks. Police arrests were associated with needle sharing and drug overdose [5]. Police abuse is so common in Russia that Russian society has coined the term police *besprediel*, or the sense that there are no limits to police power. This represents violence embedded in a social structure that perpetuates fear and terror, internalized stigma and a sense of helplessness and fatality especially among women [6]. Police violence is a problem for all PWID, but an assessment conducted in Russia suggests that police may treat women who inject drugs more harshly than men [7,8].

Women who inject drugs are marginalized and particularly vulnerable to violence. In qualitative studies in Russia on HIV and health risk, extrajudicial policing practices such as physical violence or arrests in the absence of illegal activities were commonly cited by PWID and produced fear and terror [6,9]. Consistent reports from human rights groups in Russia about police officers perpetrating sexual violence against female PWID suggest that sexual violence from police creates trauma that endures for years and contributes to women's unwillingness to seek harm reduction services [10]. An alternative report submitted by Russian civil society organizations (CSOs) to the 46th session of the UN *Commission on the Elimination of Discrimination against Women* documented sexual and physical violence perpetrated by police against women who inject drugs and trade sex [11]. The report deplored certain police practices (e.g. using drug use and prostitution offences as justification to harass or abuse women, extorting money, demanding sexual services or exposing women to physical and psychological violence).

These problems of police sexual violence against women who inject drugs and related HIV risk behaviours have not been well examined in Russia. This study aimed to determine the prevalence of sexual violence in Russia perpetrated by police against HIV-positive women who inject drugs and to evaluate potential-associated HIV risks. We also sought to understand qualitatively the phenomenon of sexual violence from police against women who inject drugs and possible strategies to address the problem, based on interviews with PWID, police and other key stakeholders working with PWID.

## Methods

We conducted a secondary data analysis of 228 women reporting drug injection using baseline survey data from the HERMITAGE study, a randomized controlled trial among 700 HIV-positive Russian drinkers testing a behavioural intervention to reduce risky behaviours [12]. We did not include men in the analysis as only one man reported sexual violence from police. The recruitment of study participants is described in detail elsewhere [11]. In brief, from October 2007 to April 2010, we recruited HIV-positive risky drinkers with reported unprotected sex in the previous six months at four HIV care and addiction treatment sites in St. Petersburg, as well as at a needle-exchange programme which referred to the treatment sites. Entry criteria included the following: age 18 years or older, HIV infection, reported unsafe sex (anal or vaginal sex without a condom) in the past six months, any risky drinking in the past six months as defined by the US National Institute on Alcohol Abuse and Alcoholism (NIAAA) [13], provision of contact information, a stable address within 150 km of the city and the ability to provide informed consent. Exclusion criteria were anticipated incarceration or intent to conceive a child. The parent trial is registered at ClinicalTrials.gov as NCT00483483.

Separately, from March through June 2012, we collected qualitative data in the form of 23 in-depth interviews with key informants to explore the phenomenon of police sexual violence in Russia and approaches to address the problem. The objective of the qualitative study component was to complement the quantitative findings by adding a variety of perspectives to explore the phenomenon of sexual violence from police through a broad range of perspectives on sexual violence from police against women who inject drugs. We recruited a variety of respondents, including PWID, police, CSO workers, and other stakeholders. Interviews were conducted by KL and FL, and a male and female research team familiar with the Russian health and addiction treatment system who are fluent in both English and Russian, have medical and anthropological training and are experienced in qualitative methodologies.

### Participant recruitment and data collection

For the quantitative study, we collected baseline data during a face-to-face survey interview with a research associate. We conducted all interviews in the Russian language. Participants were compensated 200 rubles for the baseline assessment.

For the qualitative study, we purposively recruited potentially information-rich PWID, police officers, addiction-care providers, Russian CSO workers and experts from international organizations in Russia. For that purpose, we recruited through our network of contacts of people serving PWID, asking our partners to identify and refer to us those potential study participants whom they deemed most knowledgeable about drug use and sexual violence from police. All interviews were based on a semi-structured questionnaire, conducted in the Russian language in a private location convenient for participants (who were not compensated for participation) and audio-recorded. A bilingual Russian–English speaker translated the interviews into English during verbatim transcription from audio files for analysis.

The Institutional Review Boards of Boston Medical Center and St Petersburg Pavlov State Medical University approved this study.

### Survey measures

Primary dependent variables were current (i.e. past 30 days) IDU and any reported lifetime overdose events. Receptive needle sharing in the past 30 days (i.e. having used a potentially contaminated needle that someone else had used) and the number of injections in the past 30 days were analyzed as secondary dependent variables in a sub-analysis among respondents reporting current IDU (*n*=117).

The main independent variable was sexual violence perpetrated by police, which we measured by asking the question, “Have you ever been forced to have sex with a police officer?” Although we also measured other police involvement items such as syringe confiscations (syringes are not illegal in Russia) and arrests, these were not part of the definition of the main independent variable.

Other subject characteristics of interest included age, educational status (up to primary school completion [grade 9] vs. higher), any history of incarceration, stigma scores (abbreviated Berger HIV stigma scale), depression scores (Beck's Depression Index-II), ever antiretroviral treatment, time since HIV diagnosis (under vs. over one year), risky alcohol use in the past 30 days (i.e. any as defined by the NIAAA), lifetime transactional sex (selling sex for money or drugs), incarceration, intimate partner violence victimization, childhood sex abuse victimization, suicide attempts and the number of unprotected sex encounters in the past 30 days.

### Data analysis

#### Quantitative survey

We computed descriptive statistics and applied chi-square and Student *t*-tests to describe differences in subject characteristics between groups (police sexual violence victims vs. non-victims). Separate logistic (dichotomous outcomes) and Poisson (number of injections) regression models were used to assess association between sexual violence from police and the primary (current IDU, lifetime overdose) and secondary (receptive needle sharing and injection frequency) outcomes. Potential confounders included as covariates in adjusted models were age, stigma (Berger HIV Stigma Scale), depression, childhood sex abuse victimization, history of incarceration and involvement in transactional sex. These covariates were selected based on prior literature and clinical knowledge, suggesting that these factors may confound the association between sexual violence perpetrated by police and risk behaviours. The Poisson regression model used a Pearson's chi-square correction to account for overdispersion in the data. Spearman's correlations were used to assess correlations between independent variables and covariates, and no pair of variables included in regression models was strongly correlated (*r*>0.40). We performed all analyses using SAS, applying a two-sided significance level of 0.05.

#### Qualitative

We used Nvivo 10 software [14] to code and analyze qualitative data using a content analysis approach based on theoretical memos [15]. Two coders (FL and KL) conducted multiple coding cycles based on consensus to formulate units of organization and analytic codes. We used constant comparative coding such as systematic and far-out comparisons and focused coding to identify recurrent themes and patterns [16].

## Results

### Survey

The demographics and clinical characteristics shown in Table 1 suggest that a number of risk factors and behaviours are common in this cohort of Russian HIV-positive women who inject drugs. Of note, while a higher proportion of those reporting sexual violence from police also reported involvement in transactional sex, most affected women in this cohort were not sex workers.

**Table 1:** Demographics and clinical characteristics of all HIV-positive women who inject drugs in the Russian HERMITAGE cohort stratified by history of sexual violence from police (*n*=228)

	Overall *n*=228	Reported sexual violence from police *n*=55	Did not report sexual violence from police *n*=173	*p*
Mean age (SD)	29.0 (5.4)	29.0 (4.8)	29.0 (5.6)	0.99
Education status beyond primary	123 (53.9%)	30 (54.5%)	93 (53.8%)	0.92
Incarceration, lifetime	65 (28.5%)	15 (27.3%)	50 (28.9%)	0.82
Injected drugs over 20 times in the past 30 days	87 (38.2%)	28 (50.9%)	59 (34.1%)	0.03
Stigma score (mean)^a^	24 (4.7)	24 (4.9)	24 (4.6)	0.87
Depressive symptoms (BDI-II)	179 (78.5%)	44 (80.0%)	135 (78.0%)	0.76
Ever been on ART	68 (29.8%)	14 (25.5%)	54 (31.2%)	0.42
>1 Year Since HIV Diagnosis	180 (78.9%)	47 (85.5%)	133 (76.9%)	0.17
Risky alcohol use in the past 30 days	175 (76.8%)	42 (76.4%)	133 (76.9%)	0.94
Selling Sex for drugs or money, lifetime	40 (17.5%)	18 (32.7%)	22 (12.7%)	<0.01
Victim of intimate partner violence, lifetime	185 (81.1%)	47 (85.5%)	138 (79.8%)	0.35
Childhood sexual abuse	33 (14.5%)	9 (16.4%)	24 (13.9%)	0.65
Overdose events, lifetime	164 (71.9%)	44 (80.0%)	120 (69.4%)	0.13
Any suicide attempts, past 3 months	13 (5.7%)	3 (5.5%)	10 (5.8%)	0.93
Mean number of unprotected sexual encounters in the past 30 days (SD)	19.0 (37.7)	18.7 (29.7)	19.1 (40.0)	0.93

a Berger stigma scale; higher score means more stigma.

We documented that almost a quarter (24.1%; 95% CI, 18.6%, 29.7%) of all women reported having been forced to have sex with a police officer (Table 2). The proportions reporting punitive policing practices appeared higher among victims of sexual violence than for those who were not victims.

**Table 2:** Police involvement of HIV-positive women who inject drugs in the HERMITAGE cohort, St. Petersburg, Russia (*n*=228)

Police involvement	All women *n*=228 Percentage (95% CI)	Reported sexual violence from police *n*=55 Percentage (95% CI)	Did not report sexual violence from police, *n*=173 Percentage (95% CI)
Been forced to have sex with a police officer	24.1% (18.6%, 29.7%)	n/a	n/a
Had syringes taken from you by the police	44.3% (37.9%, 50.8%)	63.6% (50.9%, 76.4%)	38.1% (30.9%, 45.4%)
Been arrested for carrying a syringe	36.8% (30.6%, 43.1%)	60.0% (47.1%, 73.0%)	29.5% (22.7%, 36.3%)
Been arrested after the police “planted” syringes or drugs on you	37.7% (31.4%, 44.0%)	50.9% (37.7%, 64.1%)	33.5% (26.5%, 40.6%)
Been forced to give money to the police to keep from being arrested	66.7% (60.6%, 72.8%)	92.7% (85.9%, 99.6%)	58.4% (51.0%, 65.7%)

Regression analyses did not show significant associations between the main independent variable reported sexual violence from police and the outcomes of current IDU, needle sharing or lifetime overdose. However, women who reported having been forced to have sex with a police officer reported more frequent drug injections (Table 3).

**Table 3:** Multivariable regression models to evaluate associations between reported sexual violence from police and dependent variables. Note that the associations with current injection drug use (primary), overdose (primary) and needle sharing are expressed as adjusted odds ratios (AOR), whereas the association with injection frequency is expressed as incidence rate ratio (IRR)

Dependent variable	Victims of police sexual violence *n*=55	Non-victims *n*=173	AOR/IRR^a^ estimate (95% CI)	*p*
Among PWID reporting ever IDU (*n*=228)				
Current IDU (past 30 days)	33 (60.0%)	84 (48.6%)	1.3 (0.65, 2.6)	0.46
Overdose (lifetime)	44 (80.0%)	120 (69.4%)	2.0 (0.94, 4.40)	0.07
Among PWID reporting current IDU (past 30 days)				
	***n* =33**	***n* =84**		
Receptive needle sharing (past three months)	18 (54.5%)	39 (46.4%)	1.26 (0.53, 2.98)	0.60
Mean injection frequency, past 30 days (SD)	73 (46)	51 (43)	1.43^b^ (1.04, 1.95)	0.03

a Logistic (binary outcomes) or Poisson (number injections) regression models adjusted for the following covariates: age, depression, stigma score, childhood sexual abuse, incarceration, selling sex in the past three months. The associations with current injection drug use (primary), overdose (primary), and needle sharing are expressed as adjusted odds ratios (AOR) [or the relative odds that the event will occur among those reporting sexual violence from police vs. those who do not], whereas the association with injection frequency is expressed as incidence rate ratio (IRR) [or the ratio of incidence rates for those reporting sexual violence from police versus those who do not]

b Represents adjusted IRR from overdispersed Poisson regression model adjusted for the following covariates: age, depression, stigma score, childhood sexual abuse, incarceration, selling sex in the past three months.

### Qualitative study

We conducted interviews in Russian with 23 participants, including 6 PWID and 3 police officers, 4 addiction physicians (narcologists), 4 workers of Russian CSOs serving PWID and 5 experts from international non-governmental organizations or international organizations in Russia. Interviews lasted between 36 and 102 min.

When asked about police sexual violence against PWID, several male PWID responded that they were not aware of such issue:

[*Is there sexual violence from police?*] No, I haven't encountered it. *Male PWID #4*

Several of those serving PWID, again predominantly males, said that they had no first-hand experience:

[*What is the interaction between police and drug users?*] Some drug addicts, essentially women who are commercial sex workers, say that they were forced to have sexual relations with the police officers. But once again, this is what I have heard several times just from drug addicts, not from the police. There are no cases in court, never. Patients don't like to discuss. *Male addiction physician #1*

Indeed, the police officers we interviewed (all male) expressed that sexual violence was a foreign concept, almost absurd to think of. This officer also pointed out that it carried a risk:

[*Is there sexual violence from police toward drug users?*] I don't know. I haven't heard about it. Haven't even thought about that. […] Before you asked this question I have never even thought about sex with a drug user. One would feel pity, disgust, even fear of AIDS. *Male police officer #1*

For others, particularly but not only female respondents, it was clear that sexual violence against women is an everyday phenomenon that particularly affects women who use drugs. Like this informant explains:

I witnessed this one instance; I'm discussing something with the police. Someone says “yesterday they brought this drug user. His wife came and begged to release him. I said let's sleep together and I'll release him. And I got laid.” And this type of stuff happens all the time, not even talking about drug users, this happened to a regular woman. But for female drug users, it's straightforward. They just say “we'll write this down, and now you'll come with us we'll do this and that. And that's it.”. […] There's no question about police sexual violence toward drug users, that's routine. If they catch a female drug user, she will 100% service them, and then they will return to business. *Male PWID #3*

While many assumed that women involved in selling sex were at particular risk of sexual violence, others pointed out that women in general and those who use drugs in particular are vulnerable. This CSO representative refers to triple stigma when she explains:

Women drug users have no protection. People feel they need to be protected from drug users. Some women are triple stigmatized, because they are drug users, they are female drug users, and they are HIV positive female drug users. *Female international expert #3*

Another Russian CSO representative reasoned that even though sex workers are more vulnerable, women who inject drugs and are not sex workers are also at risk of sexual violence from police:

[*Who do you think becomes a victim of sexual violence?*] In our city [St. Petersburg] it is mostly sex workers, because they're exposed fully and they just stand on the streets, and police can pick them up, any time, any day. But, any drug user can become a victim. Like, for example, one woman told me how even before she became a sex worker, she had this interaction with her local police officer who wanted to rape her. So, any kind of vulnerable woman can become a victim. *Female CSO staff #4*

Women who use drugs and engage in sex work may not view these abuses as “violence,” but as transactional in nature – trading sex to avoid police harassment. This CSO respondent explained that women who inject drugs and sell sex perceive sexual violence not as violence, but rather as sexual coercion being an “occupational hazard” for those who are known to sell sex:

When we were talking to a group of IDU women about this very issue of police violence, the question was basically, what sort of violence do you encounter when dealing with the police? And we talked about a few things, but it only came up LATER [*emphasis*], when we were talking about the issue of police treatment, uh … that the police sometimes coerce some sort of sexual favor to leave them alone. So it's not like they're BEATEN [*emphasis*], into submission? But it's coercion. And what was interesting was that, when I had asked the question about violence earlier, and I had used that word, “violence,” they didn't mention it in THAT [*emphasis*], context. […] So they didn't necessarily see the sexual coercion as “violence,” but more as, um, like almost … I, I don't want to say “an occupational safety hazard,” but kind of like, the cost of doing business. […] Sometimes they don't even understand that WHAT they're being subjected to can be characterized as violence. It's just so much a part of what they have had to deal with over the years they've been a sex worker or a drug user that it doesn't even register. They see violence only as being beaten. But they don't see, necessarily, the coercion of sexual services as an example of police violence. *Male international expert #5*

Another CSO representative explained how coercive arrangements of sexual violence against sex workers are apparently rooted in a former Soviet concept of volunteering labour, applying the term to a coercive, abusive “arrangement”:

Sex workers are considered “subbbotniki.” Subbbotniki is an old word, from the Soviet era, which refers to the day when you work for free. So, on Saturday [subbotain the Russian language], all the Soviet people had to work for free, for the state. And now, police see these sex workers as subbotniki. So, they serve their wishes. They are street sex workers, really poor drug users, and many of them don't have pimps, so they're really unprotected. And often, the police just comes and they say, “Okay. Now you have to work for me for free,” and they take them away and rape them. They take them away and they have to provide them sex services for free. They are pressured to provide them with free sex. But apart from free sex, they also really are abusing them. They beat them or threaten to kill them. And these people feel really unprotected because they say, “We're sex workers, we are junkies and the police can do anything with us. Even if they kill us, no one will even care, because nobody will look for us and nobody will start any kind of investigation.” So, police feel really unthreatened and they can do whatever they want. *Female CSO staff #3*

Due to the power imbalance between police and PWID, affected women have little chance to seek justice for what happens to them. Like this addiction-care provider, several respondents said that women are hesitant to disclose the problem because of an environment of mutual distrust between PWID and others in society.

Drug addicts don't like to discuss violence. Basically, they are not telling anybody, not even their doctor, who could not do anything about it anyway. There is no way to prove that they were beaten or forced to have sex with a police, it is just possible, no one would believe it coming from a drug addict. Even I am not always believing in what they're saying, they are drug addicts. *Male addiction physician #1*

Notwithstanding the different perceptions of what constitutes violence in the context of police forcing women who inject drugs to have sex with them, women (including sex workers) who have endured police sexual violence experience it as an unbearable trauma. The power imbalance between police and women seems so drastic that women who inject drugs and those who serve them hardly see any solution to the problem. This CSO representative's account also reflects the secondary trauma to the people witnessing the trauma when she recalls:

After hearing what those sex workers told me [*about the police violence they had been exposed to*], I wanted to switch off my head. For six hours I just lay in my bed, I couldn't move. It's … indigestible, you know? You can't imagine how it happens on an everyday basis. How these women are totally, absolutely powerless. They understand they can be killed, they can be raped, they can be abused in any possible way by the police officers, and nobody can protect them. Nobody can do it, you know? *Female CSO staff #3*

## Discussion

This study documents a high prevalence (24%) of sexual violence from police in a cross-sectional analysis of a cohort of Russian HIV-positive women who inject drugs. Gender-based violence against women is a global public health problem. It is a criminal justice issue and has far reaching health impact beyond immediate trauma [17]. A recent review of sexual violence globally found that more than 7% of women have ever experienced non-partner sexual violence, with a prevalence of 6.9% in Eastern Europe [18]. The proportion of women having experienced sexual violence from police in this study (24%) represents over three times the regional rate of non-partner sexual violence against women (which is not limited to police). This indicates an epidemic of sexual violence against HIV-positive women who inject drugs perpetrated by law enforcement.

This study found that women who report sexual violence from police have higher rates of punitive police involvement such as arrests and planted evidence. Sexual violence from police against women who inject drugs is associated with the risk of more frequent injections, suggesting that oppressive policing adds to the risk environment. Sexual violence is both a criminal and human rights violation. Among PWID, it carries many HIV and health risks. Due to its cross-sectional design, our study cannot infer any causality or direction of causality between violence and risk behaviours. While sexual violence from police could increase affected women's risk behaviours, the inverse might also be the case: women who are, obvious to police, using drugs and engaging in risky behaviours might be more vulnerable to their abuse and even sexual violence than those whom they do not perceive as drug users. A study conducted in Vancouver, Canada, found that PWID who experienced sexual violence in their lives were more likely to become infected with HIV, be involved in transactional sex, share needles, attempt suicide and experience an overdose [19].

The quantitative study showed that trading sex for drugs or money is not associated with women's risk of sexual violence from police. However, sexual violence from police is not limited to women who sell sex for drugs or money, albeit they are particularly vulnerable [20]. Notably the majority of women affected by sexual violence from police in our study did not report a history of sex trade. The qualitative data indicate that the sexual violence from police reported in the quantitative study may be underreported, as forced sex from police in exchange for freedom from harassment or prosecution is common and may not even be viewed as sexual violence or rape. Women do not always define these traumatic events as violence, but the trauma can be felt without that labelling. Our qualitative findings emphasize that victimization of sex workers is highly traumatizing. For women selling sex for drugs or money, sexual violence can include not getting paid for sex, sexual harassment, sexual exploitation and rape [21]. In a study of almost 900 female sex workers conducted in St. Petersburg and Orenburg, sexual coercion by police (reported by 38% of women) and rape during sex work (reported by 64%) were associated with IDU and binge alcohol use [22].

The relationship between police and women who inject drugs, particularly those involved in transactional sex, is complex, as sexual coercion can involve offers of protection from prosecution, detention or police harassments [22,24]. In this study, the police exploitation of the illegal nature of sex work, referred to as *subbotnik*, is a euphemism referring to police demanding sex in exchange for leniency towards pimps and sex workers [25]. A recent study conducted in Moscow emphasized that this practice exposes both sex workers and police officers to substantial HIV risks, as coerced sex with police is associated with increased risks of HIV and other sexually transmitted infections [26]. Our study findings add that the coercive character of *subbotnik* is based on a power imbalance between police and vulnerable women, which facilitates human rights abuse and the circle of coercion and victimization.

Our qualitative analyses indicate that that sexual violence from police is common, unchecked, and incites helplessness and trauma for women in ways that may exacerbate risky drug use, while those unaffected by the issue remain unaware, impeding their ability to serve as allies against this violence.

The qualitative data also suggest that sexual violence is under-recognized, including by male PWID, while our quantitative data indicate that the phenomenon of police sexual violence is persuasive. According to existing literature, sexual violence from police does not seem to be limited to St. Petersburg. A study conducted in other parts of Russia (Moscow, Barnaul and Volgograd) described variety of police-perpetrated violence, including extreme forms such as torture and rape, as acts of “moral” punishment of PWID and to extort confessions from them [6]. Women believed the law enforcement and legal systems to be corrupt and ineffective. Stigma, police abuse and fear of police deter women from seeking help when they experience violence perpetrated by clients or others [7]. Police sexual violence and coercion occur in other countries. In a study of over 300 women in a US drug court, 25% reported a lifetime history of sexual encounters with police. Of those women, 96% had sex with an officer on duty, 77% had repeated exchanges, 31% reported rape by an officer and 54% were offered favours by officers in exchange for sex [27].

This study's quantitative data were collected until 2010 and the qualitative data in 2012. We did not find any indications for policy or other changes in Russia that might have potentially changed our findings or conclusions. A study conducted before and after the 2011 police reforms in Russia did not observe major organizational culture changes in the police system [28].

While human rights groups have reported on the issue for some time, our findings suggest that police sexual violence represents an underappreciated human rights and public health problem. As in many settings, women affected by sexual violence in Russia can be highly stigmatized. This study's qualitative findings indicate that this stigmatization is much more likely for women who use drugs and/or have HIV. Concealment of sexual violence from police by affected women because of the associated stigma limits awareness about this health and human rights problem, even among male peer PWID and domestic and international organizations. This lack of awareness perpetuates the vicious cycle of vulnerability and victimization. In this complex context, several stigma identities related to HIV infection, drug use and sex work might interact. To mitigate these adversities, raising social awareness and empowering affected women might strengthen their resilience and protect them from violence. The larger restrictive drug policy environment and structural factors such as lack of accountability, criminalization of drug use and sex work that create the ground for discrimination and sexual violence, even when not perceived as such, urgently require larger reforms [29–30].

Not only female victims are exposed to risks. Police officers who have sex with HIV-positive women expose themselves and their sexual partners to an increased risk of HIV transmission. Sexual violence from police against women, assessed in US drug courts, involved unprotected sex in for almost half of the women (49%) [26]. Police training needs to raise awareness for victims’ human rights violations and traumatization, and also for HIV risks for perpetrators. Framing HIV risks in an occupational health context has been shown to increase risk awareness in the United States and Kyrgyzstan [32–33].

## Limitations

The quantitative aspect of this study was observational in design and thus limited in its ability to assign causality or ascertain the directionality of the observed association between police sexual violence and injection frequency. While sexual violence from police might lead women to inject more often, reverse causality is likewise conceivable. Those who inject more frequently are more likely to be exposed to police and might be more vulnerable to victimization or less likely to resist sexual violence. More research on the causality of the observed associations and their mechanisms is needed.

For our qualitative study, we recruited a broad range of respondents, which limited out ability to explore in depth the perceptions of particular respondent groups. Our qualitative data are narratives from respondents willing to talk to us, and we were limited in our ability to directly interview perpetrators and victims.

## Conclusions

Sexual violence perpetrated by police against women who inject drugs in this cohort of HIV-positive Russians is unacceptable and warrants further study and intervention. Taken together, quantitative and qualitative data suggest a potentially pervasive sexual violence by police against women who inject drugs that is largely unrecognized by male PWID and others who are not directly affected. In this study of HIV-positive women with current IDU, sexual violence from police was associated with more frequent IDU. These findings implicate sexual violence as adding to the risk environment of HIV-positive women who inject drugs.

Sexual violence from police represents an under-recognized human rights and public health problem, and policy efforts reacting to this evidence are urgently needed. These forms of sexual violence have far-reaching health and social consequences. Raising social awareness and calling and exposing episodes of sexual violence from police for the criminal and human rights offences that they are, are crucial to reengineering the culture that currently condones this. Furthermore, interventions are needed to build resilience among affected women, protect them from violence and reduce HIV transmission that follows from sexual violence from police.
